# Some things change fast, other take their own sweet time

**DOI:** 10.4103/0971-3026.63038

**Published:** 2010-05

**Authors:** Bhavin Jankharia

**Affiliations:** Editor-in-Chief, The Indian Journal of Radiology and Imaging “F”, 1^st^ Flr, Bhaveshwar Vihar, 383, Sardar V P Road, Mumbai-400 004, India. E-mail: editor@ijri.org

Dr. ML is a musculoskeletal and an MRI radiologist working in a Mumbai Hospital. She is also a popular speaker. A few days before the recent January IRIA, she was asked to review some of the educational and scientific exhibits that had been submitted for inclusion in the conference. She came across one particular exhibit on MRI of the knee, which seemed a bit familiar. As she went through the exhibit, which was from a post-graduate DNB student studying in a hospital in Hyderabad, she realized that the entire exhibit was based upon the images stolen from one of her own MRI knee lectures. She was very upset and called the head of the radiology department of that hospital, who said that he was an honorary and could not really do anything. Eventually, no one was willing to take responsibility for this and the student got away scot-free. Essentially, he is a thief, but a white-collar one and so walks free.

A few months ago, we picked up another attempt at plagiarism, by a post-graduate student from Chennai. Since I knew his head of department, I e-mailed him separately to let him know what his post-graduate student was upto. I never received a reply from him, but the student e-mailed me a month later asking me what I thought of myself and how dare I go behind his back to his head of department. He then sent another e-mail saying that our IJRI is anyway a trash journal and that he had managed to get the same plagiarized article published in another “better” journal. Again, no one has bothered really to take action against him and the student is getting away with robbery.

We started our crusade against plagiarism in June 2007 and since then the number of “lifted” articles has reduced quite a bit. We still however get “copy-pasted” articles from students who when asked for explanations invariably apologize saying that they did not know they were not supposed to lift. Rarely, like the Chennai student, someone will argue.

Why don't students and radiologists realize that “copying” is unacceptable and that to take someone else's work and to use it without permission is as good as stealing? Why do students think it is acceptable to shoot lecture slides with digital cameras without permission? Why do organizers of conferences think it is fine to shoot videos of speakers without their permission and to make DVDs for public circulation? When will we learn the concepts of copyright and of asking for permission?

Maybe once upon a time in our country, none of this mattered. Today, all of this is important. The times are changing.

Also, in these changing times, the practice and business of radiology are changing in other arenas as well. We have two very interesting articles in this issue that must be read by all radiologists. The one that is likely to create quite a flutter in the next few months is an article on NABH for radiology by Dr. Zaidi from the Quality Council of India. Accreditation is soon to become a reality that radiology practices will have to face. As healthcare becomes more and more organized, it is necessary to not only have basic minimum standards, but also adopt practices and processes that allow us to achieve the topmost standards of quality care.

The other article is on OSCE, i.e., objective structured examinations in radiology, by Dr. Anurag Agrawal from the National Board of Examinations. OSCE is a reality in many other disciplines and its adoption in radiology will help to overcome examiner bias and other issues that tend to make examinations subjective and prone to errors and mistakes.

Hopefully, with the adoption of these measures as well as other processes in the years to come, the face of radiology will change for the better in our country.

